# Hospital Book-Keeping and Accounts

**Published:** 1890-03-15

**Authors:** 


### HOSPITAL BOOKKEEPING AND ACCOUNTS.

The forthcoming volume of *Burdett's Hospital Annual* for 1890, will contain a complete set of books for hospitals, which it is hoped will prove of service to the treasurers, secretaries, and others engaged in their management. Mr. Burdett's system includes a Glossary of all the items used in hospitals which have to be analysed and brought to account in the books throughout the year. Everyone interested is invited to test the Glossary by asking, as Mr. Lloyd, J.P., asked at the meeting, under what head any particular article they may mention should be posted in the books. It is hoped that very many treasurers, secretaries, matrons, and others will test the Glossary in this way so as to make it as perfect as possible. The editor will be glad to present a free copy of the *Annual* to the three correspondents who mention the greatest number of items not already included in this Glossary. All letters on this subject should be addressed to the editor of *The Hospital*, and have the word "Glossary" written distinctly in the left hand corner of the envelope.

